# Consucrats and Pathocrats: The Prequel, Quel, and Sequel; A Response to the Recent Commentaries

**DOI:** 10.34172/ijhpm.2021.124

**Published:** 2021-09-06

**Authors:** Evelyne de Leeuw

**Affiliations:** ^1^Centre for Health Equity Training, Research and Evaluation (CHETRE), University of New South Wales, Sydney, NSW, Australia.; ^2^South Western Sydney Local Health District Population Health, Liverpool, NSW, Australia.; ^3^Ingham Institute for Applied Medical Research, Liverpool, NSW, Australia.

 To recapitulate: I wrote a piece critical of ‘consumer’ involvement and representation mechanisms in the medical-industrial complex. In that piece, I coined the term ‘consucrat.’ Consucrats, I suggested, are “*a volunteer channel of the voice of the receiving ends of healthcare procedures and policies; embedded in the system they have grown to become co-opted apparatchiks who may rhetorically claim to speak truth to power, but may no longer be the representative voice of ‘the consumer.’*”^1^

 I proceeded by outlining how the key values of consumer action in health are challenged and challenging. These crystallise around designation, professionalization, and representation. I suggested that a firmer and more critical foundation for action of people being enabled to influence and improve not only health service delivery, but more broadly, the determinants of health, is important.

 The editors of this journal then invited comments on what I thought might have been perceived as a slightly exotic or esoteric analysis. The importance of user representation in any system is considered an immutable article of faith. My ranting might have been seen as the sales pitch of a raincoat saleswoman in the desert. But fortunately O’Donovan,^2^ DeCampet al^3^ and Keeling^4^ were gracious enough to think and argue along with me.

 They all challenged, to some extent, my framing of the phenomenon, and my neologising the consucrat. They not only criticised the notion for it being too derogatory^2^ (in line with the femocrat and abocrat), but also suggested an expansion of my casting. Keeling extends the argument and proposes the importance of an analysis of the ‘profecrat’ – professional service representatives.^4^ She nicely applies the triad of designation-professionalisation-representation to complex service ecosystems. It did make me realise that the consucrat only exists – and is legitimised – by the grace of other parts of the ecosystem, be they structures, people, or institutions.

 And indeed, there is a very sound argument to be made – and a great deal to be learned – from such ecosystems. Both Keeling and O’Donovan provide strong pointers. Critically, with DeCamp et al,^3^ the issue of power is prominently presented. Power, of course, is critical in understanding how systems develop and maintain themselves.^5^ I have made that argument earlier in an assessment of the feasibility of thriving Health in All Policies.^6^ It does not mean that these and similar lessons^7^ are heeded, though. There is still much to be appreciated and transferred from these ‘service ecosystems’ into the healthcare delivery advocacy and enhancement.

 For one, a small body of literature is emerging about the health promotion dimensions of unionisation.^8,9^ Although there is a general lament that the golden age of trade and labour unions is over, and new modi operandi need to be forged for worker representation in the era of the gig economy,^10^ if there is any service ecosystem that has a long standing track record in militant, activist and empowered representation, it is the union movement. Whether we call them consucrats or profecrats, I think much can be learned from the rise and fall of unionism in advocacy and bargaining.

 Part of the effectiveness (and nature of true representation) indeed is agency of actors – and the mastery of the critical tools of agency: language and semiotics. I was pleased (and philosophically a bit dumb-founded, I admit) to see O’Donovan’s^2^ reference to the work of Donna Haraway, and in particular to her casting of the Chthulucene^11^ as a vast, interconnected and intersectional environment. O’Donovan capably applies this tentacled notion to a world of pandemics, healthcare system exuberance and collapse, and planetary climate change crises. Coming from a perhaps more optimistic health promotion background I prefer the metaphor of the ‘rhizome’ pervading any and all^12^ – it paints a slightly less catastrophic picture. But urgency is what we need – and a slow growing connected rhizome may not be the right metaphor.

 And this is, I guess, what the debate boils down to. I have covered the prequel to this episode and need to set up you, the audience, for the sequel. Where are the consucrats, profecrats, and O’Donovan’s cosmedics^2^ heading?

 I spent many years thinking about my consucrat argument (in fact, since I edited a book^13^ with Hans Löfgren and Michael Leahy on the role of the consumer health movement in health policy development – to which O’Donovan contributed a chapter). The crux of my concern was that patients, healthcare users, and anyone exposed to the (social, commercial, political) determinants of health have become accomplices in the maintenance of the medical-industrial complex and the perverse effects of its components.^14^ That is, that people and/in their environments somnambulate into ever greater exploitation by a, yes let’s call it, Chthulucene. An epoch that uncritically disembodies people as simple elements in a very large perverse planetary mechanism that ultimately is not aiming for health.

 For instance, for decades now I have been stunned by the term ‘patient-centred care.’^15^ It implies that such a novel gaze complements or critiques ‘disease-centered care’ or worse ‘physician-centered care.’ What? Really? Such ‘cares’ exist? Yes – simply by not naming them they do exist. Linguistic semantics suggest that issues and phenomena only come into being by crafting their sounds or names. The fields of politics, health and medicine seem particularly rich bodies of linguistic ore.

 With the three commentaries and the consucrat piece we have started to develop a language to describe how to precisely ‘enable individuals, groups and communities to take control over the determinants of health, and thereby improve their health.’^16^ This goes beyond simplistic notions about empowerment, consumers, and patients. It involves Chthulucenes, profecrats, cosmedics, consucrats, and pathocrats. You haven’t heard of the latter? Then – *give* it meaning!

## Ethical issues

 Not applicable.

## Competing interests

 Author declares that she has no competing interests.

## Author’s contribution

 EdL is the single author of the paper.
